# The Role of the Nasal Cavity in the Pathogenesis of Prion Diseases

**DOI:** 10.3390/v13112287

**Published:** 2021-11-16

**Authors:** Anthony E. Kincaid

**Affiliations:** Departments of Pharmacy Sciences and Medical Microbiology and Immunology, Creighton University, 2500 California Plaza, Omaha, NE 68178, USA; akincaid@creighton.edu

**Keywords:** PrP^Sc^, TSE, prions, nasal cavity, prion pathogenesis, olfactory system, prionemia, centripetal, centrifugal

## Abstract

Prion diseases, or transmissible spongiform encephalopathies (TSEs), are a class of fatal neurodegenerative diseases caused by the entry and spread of infectious prion proteins (PrP^Sc^) in the central nervous system (CNS). These diseases are endemic to certain mammalian animal species that use their sense of smell for a variety of purposes and therefore expose their nasal cavity (NC) to PrP^Sc^ in the environment. Prion diseases that affect humans are either inherited due to a mutation of the gene that encodes the prion protein, acquired by exposure to contaminated tissues or medical devices, or develop without a known cause (referred to as *sporadic*). The purpose of this review is to identify components of the NC that are involved in prion transport and to summarize the evidence that the NC serves as a route of entry (centripetal spread) and/or a source of shedding (centrifugal spread) of PrP^Sc^, and thus plays a role in the pathogenesis of the TSEs.

## 1. Introduction

Animals that naturally acquire TSEs, including deer, reindeer, elk, moose, and sheep, have a prominent snout and a well-developed sense of smell. Like many species, they actively use their nose to explore their environment for spatial orientation purposes, social communication, the detection of predators, foraging for food, and the selection of potential mates, and they rely upon their memory of previous olfactory experiences to respond appropriately [1]. Prions can persist in the environment for years and have been detected in several bodily excretions including saliva, feces, urine, and blood and in decaying carcasses, placentas, soil, and plants [2,3,4,5,6,7,8,9,10,11,12,13,14,15,16,17]. Thus, it is reasonable to expect that animals expose their nasal cavities (NCs) to PrP^Sc^ via direct contact with infected animals or following the inhalation of prions shed in the environment and that the NC may serve as an entry point for animal prion diseases. Moreover, given the demonstration of PrP^Sc^ in a variety of animal bodily excretions, it is possible that prions are shed in nasal secretions from affected animals.

Humans also have a well-developed sense of olfaction but are not as behaviorally dependent upon their sense of smell to interact with the surrounding world, and to date, there have been no reports of neuroinvasion via inhalation into the NC of humans [1,18,19]. However, there is evidence for prions binding to dust collected from scrapie-affected farms and speculation that the olfactory bulb might serve as an entry site for prion neuroinvasion based on the involvement of olfactory structures in animal and human prion diseases [20,21,22,23,24,25]. The purpose of this article is to summarize the evidence for the centripetal and centrifugal spread of prions via the NCs of animals, including humans, and to summarize the role of the NC in prion pathogenesis.

## 2. Functional Organization of the Nasal Cavity and Prion Pathogenesis

**Figure 1:**
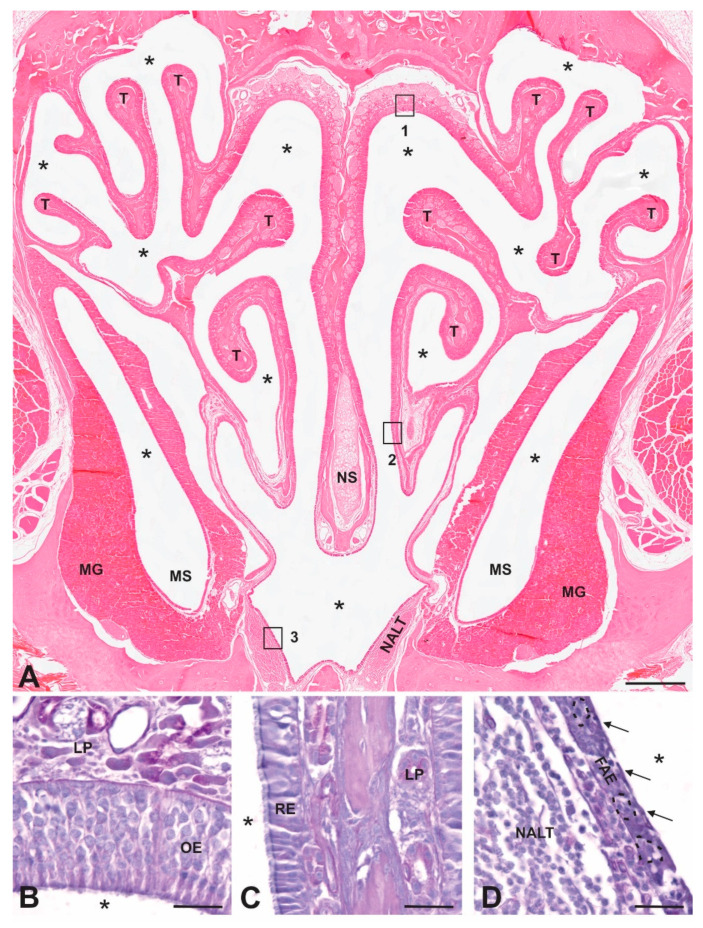
Anatomy of mammalian nasal cavity. (**A**) H&E-stained coronal section of a decalcified hamster nasal cavity showing the bony and cartilaginous nasal septum in midline (NS) and the turbinate bones (T) that increase contact between inhaled material and the epithelia that line the cavity. Note the presence of extensive mucus glands (MGs) in the lamina propria surrounding the maxillary sinus (MS) and the unencapsulated nasal associated lymphoid tissue (NALT) in the floor of the NC. Asterisks mark airspaces. Boxes 1–3 are enlarged in panels **B**–**D**, respectively. (**B**) Olfactory epithelium (OE) lines the upper part of the NC and contains olfactory sensory neurons with dendrites that extend into the mucus layer of the NC airspace and axons that collect into nerve fascicles that form the olfactory nerve and project directly into the olfactory bulb of the brain. (**C**) Respiratory epithelium (RE) contains a single layer of pseudostratified ciliated columnar epithelial cells that overlay a loose connective tissue layer, the lamina propria (LP), that contains mucus glands and blood vessels. (**D**) Follicle-associated epithelium (FAE) contains microfold cells (M cells; indicated by arrows) that transport antigens in the NC to underlying lymphatic cells in intraepithelial pockets (dotted lines) and the nasal associated lymphoid tissue (NALT). Scale bars: A = 400 µm; B, C, and D = 20 µm.

**Figure 2:**
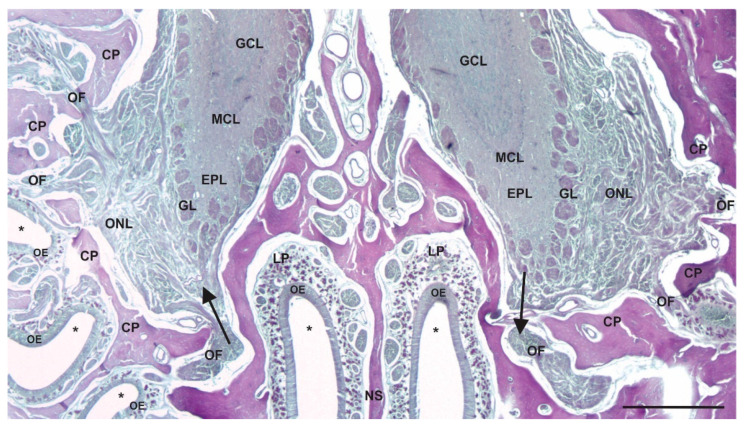
Route for centripetal and centrifugal transport between the olfactory epithelium and olfactory bulbs. A decalcified coronal section of the bony cribriform plate (CP) of the skull, the bony nasal septum (NS), and the superior part of the NC stained with periodic acid–Schiff reagent (asterisks indicate airspace of the NC). The olfactory nerve fascicles (OFs) pass through holes of the CP connecting the olfactory sensory neurons of the olfactory epithelium (OE) to the glomeruli (GL) of the olfactory bulbs in the CNS. The axons of the olfactory sensory neurons collect into nerve fascicles in the lamina propria (LP) of the superior NC. Note the relatively short and direct route these axons take from the epithelium to the brain. The arrow on the left indicates the direction of centripetal spread, while the arrow on the right indicates the direction of centrifugal spread. GCL = granule-cell layer; MCL = mitral-cell layer; EPL = external plexiform layer; GL = glomerular layer; ONL = olfactory-nerve-fiber layer. Scale bar = 400 µm.

The mammalian NC is an irregularly shaped airspace within the skull and is the initial segment of the respiratory tract. The mucosae that line the NC are specialized for conditioning inhaled air by heating, moistening, and cleaning it and facilitating the sense of olfaction. The walls, floor, roof, and medial septum consist of bone and hyaline cartilage, and the lateral walls have irregular projections of bone, known as conchae or turbinates (Figure 1). The shape, size, and number of these thin bony plates vary between mammals, but in each case, they function to increase the NC surface area and turbulence of the inhaled air, both of which facilitate contact between the inhaled material and the nasal mucosa. The bony and cartilaginous surfaces are lined superficially by epithelial cells that sit on a loose connective tissue layer, the lamina propria, that contains numerous mucosal glands, blood and lymphatic vessels, and nerves (Figure 1). The abundance of mucus glands and blood vessels in the lamina propria is important in moistening and warming inhaled air. There are several types of epithelia that line the surface of the NC, each with morphological features that mediate specific functions, and while different species have different proportions of epithelial types, overall, there is a similar organization. Anteriorly, the vestibule of the NC is lined by a stratified squamous epithelium that is specialized for resisting abrasive forces. Posterior to the vestibule, the inferior portion of the NC is lined predominantly by respiratory (non-sensory) epithelium, which consists of pseudostratified ciliated columnar epithelium interspersed with mucosal goblet cells (Figure 1). This type of epithelium functions to trap particulate airborne matter in mucus and move it towards the oropharynx via the coordinated movement of cilia. The olfactory (sensory) epithelium is located predominantly in the superior portion of the nasal cavity and contains the olfactory sensory neurons (OSNs) that mediate olfaction (Figure 1). The dendrite of OSNs extends towards the surface of the epithelium and expands to form a knob, which contains a variable number of cilia that extend into the mucus layer of the NC. The cilia contain receptors that bind odorant molecules and transduce odors into electrical signals [26]. Unmyelinated axons of the OSNs project from the base of the cells and collect to form fascicles of the olfactory nerve (ON), cranial nerve I, which projects through openings in the skull, the cribriform plate, to synapse in the olfactory bulb (OB) in the brain (Figure 2). These neurons provide the most direct synaptic connection between a surface of the body, the nasal mucosa, and the central nervous system (CNS) and are vulnerable to inhaled pathogens, providing a potential direct pathway into the brain [27]. An interesting feature of most mammalian OSNs is their continued turnover (the average lifespan is 30–90 days under normal circumstances), with ongoing neurogenesis and continual shedding into the periphery, properties not shared with other neurons reviewed in [28,29]. Terrestrial vertebrates have an accessory olfactory structure in the NC, the vomeronasal organs (VNOs). These are paired, blind-ended tubes located in the ventral anterior part of the septum of the NC and contain sensory neurons termed vomeronasal sensory neurons (VSNs) that project their axons into the accessory olfactory bulb. The VNO mediates several aspects of reproductive behavior by detecting and relaying chemosensory information from the environment into the CNS [30].

In support of the potential for the NC to serve as a direct route of entry for inhaled prions to the brain is the observation that olfactory, trigeminal and autonomic nerves that innervate structures in the NC transport some viruses directly to the brain [31,32,33,34,35,36,37,38]. Additionally, the normal isoform of the prion protein (PrP^C^), which is required for the transmission of prion diseases, is expressed by OSNs of the olfactory epithelium, providing further support for the potential for the direct neuroinvasion of PrP^Sc^ following inhalation [39,40,41].

Additional potential contributors to prion entry in the NC are specialized lymphoid structures, the nasal associated lymphoid tissue (NALT). The NALT is unencapsulated collections of lymphocytes and follicular dendritic cells organized into follicles, covered by a follicle-associated epithelium (FAE) that contains microfold (M) cells and is in the ventral NC (Figure 1). Animal species that are experimentally and naturally affected by prion diseases, including mice, hamsters, rats, sheep, cattle, and primates, are known to possess NALT [42,43,44,45,46,47,48,49,50]. There are two features of the NALT that support the potential for prion entry. First, M cells are known to transport antigens, including bacteria and viruses, from the airspace of the NC to the underlying lymphoid cells of the NALT, and second, the lymphoid cells of the NALT express relatively high levels of PrP^C^ [44,51,52,53]. It should be noted that the M cells found overlying the NALT are morphologically and functionally similar to the M cells of Peyer’s patches in the gut reviewed in [54]. The ringed arrangement of the pharyngeal tonsils, palatine tonsils, lingual tonsils, and tubal tonsils surrounding the junction of the naso- and oropharynx is known as Waldeyer’s ring; these lymphoid structures are the first to encounter inhaled or ingested pathogens and are thought to be a human equivalent to NALT [55].

## 3. Experimental Evidence for the Role of the NC in Prion Entry following NC Exposure

### 3.1. Efficacy and Efficiency of the NC Route of Entry

There have been multiple demonstrations that PrP^Sc^ inhaled into the nasal cavity causes disease (see Table 1). These studies have been carried out in hamsters [56,57,58], mice [58,59], transgenic mice [60], sheep [61] and deer [62,63] using different strains of prion-infected brain homogenate (bh) [56,57,58,61] or aerosolized prions [59,62,63]. The inoculation of the NC with infected bh or aerosolized prions resulted in disease in all the animals in these studies with one exception. Hamsters inoculated with the DY strain of transmissible mink encephalopathy (TME), which does not replicate in the peripheral lymphoreticular system, did not develop disease [58,64]. It is worth noting that DY-TME is transported to LRS tissues, and inoculum can be detected, but the replication of prions cannot be detected [64]. In a study where a comparison was made between oral and NC routes of inoculation, the two routes were determined to have similar incubation periods, but the NC route was determined to be 10–100 times more efficient [56]. The increase in efficiency was corroborated in a study of the aerosol transmission of chronic wasting disease (CWD) in white-tailed deer [62]. Thus, a lower dose of inoculum inhaled into the NC caused disease when compared to the ingestion of an infected food pellet. The difference in efficiency between the two routes of infection may be due to the relatively harsh environment of the gut, where alimentary fluids have been shown to markedly degrade prions in sheep [65,66]. It is possible in both natural and experimental settings that oral and nasal cavities are exposed to prions as animals explore the environment through sniffing and/or licking and take in relatively large amounts of infectious material through repeated exposures of both cavities; therefore, both routes could be involved in the entry of prions into the body of a given animal.

### 3.2. Mechanisms of Transepithelial Transport and Neuroinvasion following NC Exposure

For prions to enter the body via the NC following inhalation, they must move from the airspace across the nasal mucosa. Candidate entry mechanisms include direct uptake and transport by OSNs or VSNs, transport across or between olfactory or respiratory epithelial cells, and M-cell transport across the FAE. As discussed earlier, there are examples of the direct neuroinvasion of the brain via the ON by some substances. However, evidence to identify the transport of PrP^Sc^ as centripetal spread (entry into the CNS) versus centrifugal spread (transport from the CNS to peripheral structures) via the ON requires the demonstration of PrP^Sc^ in the OM and ON prior to the appearance of infectious prions in the OB and AOB. The OB and AOB are the synaptic targets of OSN and VSN axons, respectively, and the demonstration of PrP^Sc^ in either of these two structures prior to detection in the OSNs or VSNs could be due to the centrifugal spread of prions. To differentiate the centripetal spread from the centrifugal spread of prions requires the collection and analysis of NCs throughout the course of the incubation period, and not just the analysis of tissue collected after clinical signs have appeared and PrP^Sc^ has likely spread throughout the CNS. There are no reports of PrP^Sc^ in OSNs or the ON prior to their detection in the OBs in those studies that have collected tissues, including NCs, prior to the onset of clinical signs [56,57,58]. The apparent lack of centripetal spread of prions via the ON following inhalation into the NCs of experimental animals is supported by reports in natural hosts. In one study, prions were not detected in either OSNs or the OM in 24 cases of naturally occurring scrapie, and in another study, there was a lack of prion detection in the OM and the OBs of white-tailed deer inoculated with prions complexed to montmorillonite clay 175 days post-inoculation [23,63]. The reason for the apparent lack of centripetal spread of PrP^Sc^ via the ON is not known but may be the relatively low or uneven expression of PrP^C^ in OSNs [39,40]. In summary, to date, there is no evidence for the centripetal spread of PrP^Sc^ into the CNS via uptake and transport in the ON following inhalation into the NC. However, the centripetal spread of PrP^Sc^ via OSNs or the ON cannot be ruled out definitively, as it is possible that the expression or accumulation levels of PrP^Sc^ following NC exposure are below the level of detection of immunohistochemistry (IHC).

Other potential routes of neuroinvasion in the NC include transport via the trigeminal nerve and autonomic nerves known to innervate the nasal mucosa. Free nerve endings of the trigeminal nerve are located within the epithelia of the NC and detect somatosensory stimuli such as touch, pain, and temperature [67]. These peripheral fibers from the ethmoid and nasopalatine branches of the trigeminal nerve course between epithelial cells of the nasal mucosa but generally do not reach the airspace of the NC, terminating deep to the tight junctions that connect adjacent epithelial cells [68]. More recently, a population of solitary chemoreceptor cells was identified scattered across the nasal mucosae of rats and mice [69]. These cells express α-gustducin, similar to taste cells on the tongue, and respond to bitter substances; they are thought to mediate protective reflexes such as sneezing, coughing, or apnea in response to inhaled toxins [69,70]. These chemosensitive cells extend to the surface of the nasal mucosa and are innervated at their base by peripheral branches of the trigeminal nerve, which puts them in a position to be involved in the neuroinvasion of inhaled prions. Other nerves located in the NC that could potentially transmit prions into the brain are parasympathetic and sympathetic fibers that innervate mucus glands and blood vessels, respectively. The transport of prions via these autonomic fibers could be similar in some respects to the route of neuroinvasion from the gut wall following per os exposure as reported by McBride et al. [71]. However, there have been no reports of prions associated with these nerves, or their respective ganglia, following NC exposure. PrP^Sc^ has been detected in the spinal trigeminal tract and nucleus, and which are brainstem targets of trigeminal sensory neurons, as early as 100 days after inoculation into the NCs of hamsters (60% of the incubation period). This time point, in the middle of the course of the incubation period, makes it difficult to ascertain if the nerve is a source of the centripetal spread of prions, or if these brain areas are involved via connections with other affected brain areas [57]. There have been no studies that have examined trigeminal ganglia for the presence of PrP^Sc^ after the inhalation of infected bh into the NC to determine if they are involved in the centripetal spread of prions.

There is evidence for the transepithelial transport of inhaled PrP^Sc^ via M cells of the FAE that overlie the NALT and for the paracellular transport of PrP^Sc^ between cells of the respiratory epithelium, olfactory epithelium, and FAE [72]. These examples of transepithelial transport in the NC are observed in hamsters and mice 15–180 min following inhalation [72,73]. PrP^Sc^ was detected within a small number of M cells in the FAE overlying the NALT; this type of transepithelial transport was previously noted in an in vitro model of M-cell transport [74]. Further support for the role of M cells in PrP^Sc^ transport comes from studies that showed that M-cell depletion blocked PrP^Sc^ deposition in Peyer’s patches, neuroinvasion, and disease development following oral exposure [75]. The paracellular transport of inhaled infected and mock-infected bh was observed in nearly all the animals (hamsters and mice) between epithelial cells of the NC and was neither strain-specific nor an artefact of gas anesthesia [72,73]. In these same animals, PrP^Sc^ was identified in the lumens of lymphatic vessels in the lamina propria within minutes of the inhalation of prions. The rapid transport of PrP^Sc^ across the nasal mucosa and entry into lymphatic vessels is consistent with the early detection of PrP^Sc^ in the blood of hamsters and deer that was reported to begin within 15 min following inhalation [76,77].

### 3.3. The Role of LRS Tissues in Prion Pathogenesis following NC Exposure

PrP^Sc^ has been detected in various LRS tissues following the inhalation of prion-infected bh or aerosol into the NC (Table 1). The LRS tissues that have been examined and found to have PrP^Sc^ include lymph nodes [56,58,61,63], tonsils [61,62,63], spleen [56,58,59] Peyer’s patch [56,63], rectoanal mucosa-associated lymphoid tissue (RAMALT) [62], and NALT [56,57,58]. In some of these studies, the tissues were not collected from animals until after the onset of clinical signs of disease, so it was not possible to determine if the deposition of PrP^Sc^ preceded, coincided with, or followed neuroinvasion [58,59,61]. In those studies where tissues were collected from animals at various time points prior to the onset of clinical signs, a temporal analysis of PrP^Sc^ deposition in the LRS tissues could be performed [56,57,62,63]. The NALT was the first LRS tissue that was identified to contain PrP^Sc^ in two studies, as early as 20% of the incubation period in one study [56] and at 50% of the incubation period in another [57]. PrP^Sc^ was detected in the tonsils at 25% and in the RAMALT at 50% of the incubation period following aerosol transmission to white-tailed deer [62]. In a separate study, PrP^Sc^ was detected in the lymph nodes of white-tailed deer at 3 months following the aerosol transmission of prions bound to montmorillonite clay [63]. In each case, PrP^Sc^ was detected in LRS tissues prior to detection in the nervous system, similar to what has been reported when PrP^Sc^ was ingested via the oral route [78].

Regarding the involvement of the NALT in the pathogenesis of PrP^Sc^ entry via NC exposure, it is not clear if prions are transported directly to the NALT following inhalation, or if they reach the NALT via the systemic prionemia that follows the inhalation of prions [76,77]. Regardless of how PrP^Sc^ reaches the NALT, it proceeds to progressively accumulate over time, until deposits fill the structure [56]. It is also not clear if there is a mechanism for the transport of PrP^Sc^ from the NALT into the CNS, but sympathetic post-ganglionic fibers have been identified within the NALT that might be a source of neuroinvasion [79]. The cell bodies of these fibers are located in the superior cervical ganglion [79] and are synaptically contacted by sympathetic preganglionic fibers that originate from sympathetic preganglionic neurons located in the intermediolateral cell column of the rostral thoracic spinal cord. As mentioned earlier, there have been no reports of PrP^Sc^ in the superior cervical ganglion following the inhalation of prions into the NC.

The ability of inhaled prions to cause disease in mice that lack a functional LRS has led to speculation that PrP^Sc^ can directly infect the brain via the ON, without a replication phase in lymphoid organs [58,59], but as discussed previously, there is no evidence for the centripetal spread of prions via ONs following inhalation into the NC. The route of neuroinvasion from the NC in these immunodeficient mice remains to be determined.

## 4. Evidence for the Presence and Shedding of Prions in Nasal Mucosa and Secretions

### 4.1. Experimental Evidence

**Figure 3:**
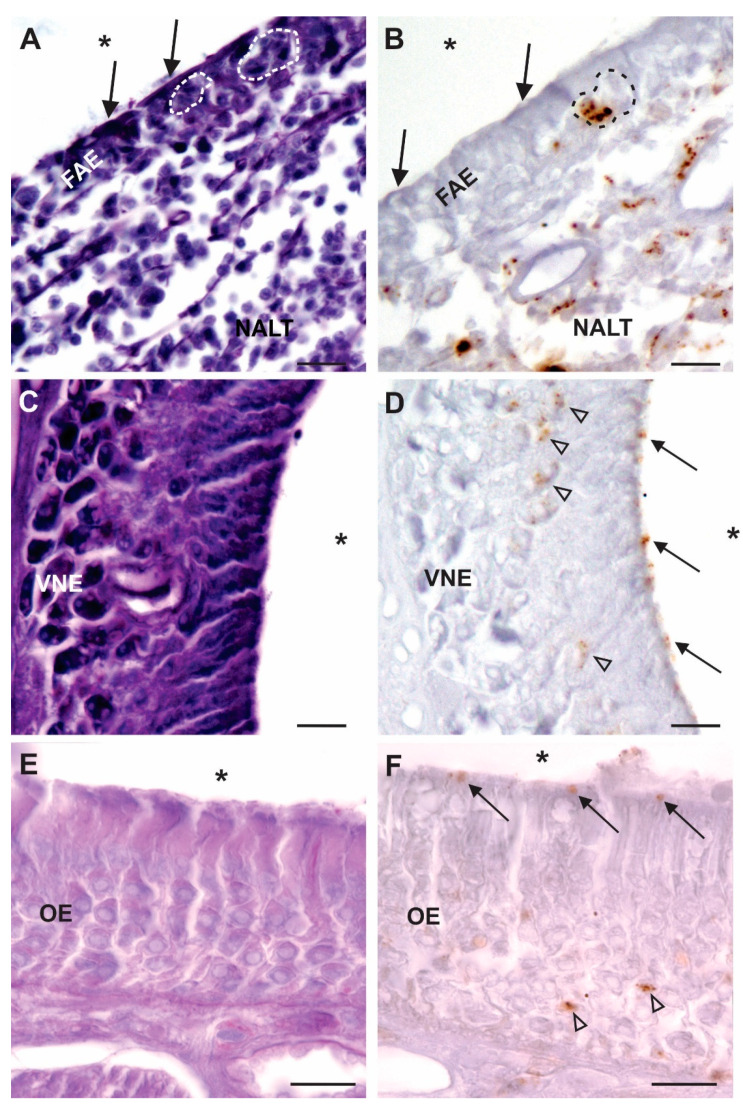
PrP^Sc^ in the NC of clinically ill hamsters following inhalation of prions into the NC (asterisks indicate airspace of the NC). (**A**,**C**,**E**) Periodic acid Schiff-stained sections, and (**B**,**D**,**F**) near-adjacent immunohistochemically processed tissue sections for the presence of PrP^Sc^ using the 3F4 antibody. (**A**,**B**) PrP^Sc^ in intraepithelial pockets (indicated by dotted lines) of the follicle-associated epithelium (FAE) and the underlying nasal associated lymphoid tissue (NALT). (**C**,**D**) PrP^Sc^ in the vomeronasal epithelium (VNE), in both the cell bodies (indicated by arrowheads) and apical surfaces of the sensory neurons (indicated by solid arrows). (**E**,**F**) PrP^Sc^ can be seen in the OE, in the OSN cell bodies (indicated by arrowheads), and in dendrites at the apical surfaces of the cells (indicated by solid arrows). Scale bars = 10 µm.

As mentioned previously, OSNs are continuously generated and shed and therefore represent a mechanism for shedding PrP^Sc^ into the environment. PrP^Sc^ has been detected in the NC of animals who have demonstrated clinical signs of prion disease (Table 2). Prions were detected in OSNs of the OM and VSNs of the VNO in the NCs of hamsters that were inoculated intracerebrally or directly into the OB with HY-TME [80,81]. Transport of PrP^Sc^ from the CNS to the OSNs and VSNs in the NC are examples of centrifugal spread of prions, but in these experiments the inoculations, made directly into the cerebrum or OBs, did not model natural routes of exposure and may actually have involved transport by axons damaged by the inoculation (in the case of OB inoculations). To determine if the centrifugal spread of prions to the NC could occur in animals following a peripheral, and potentially natural, route of exposure we examined the NCs of hamsters who inhaled HY-TME bh into their NC and later demonstrated clinical signs of disease. Examination of their NCs demonstrated PrP^Sc^ in OSNs of the OM, VSNs of the VNO and in cells located in the FAE and NALT, indicating centrifugal spread of PrP^Sc^ and the potential for the shedding of infectious prions in nasal secretions, albeit at the end stage of the clinical course (Figure 3). Infectivity in the nasal mucosa collected from cattle terminally ill with bovine spongiform encephalopathy (BSE) was determined using transgenic mouse bioassay, although PrP^Sc^ was not detected in the nasal mucosa using immunohistochemistry or protein misfolding cyclic amplification (PMCA) [82]. In two separate field studies CWD prions were detected in nasal brushings that were collected antemortem from farmed white-tailed deer, and farmed and free ranging Rocky Mountain elk, using real-time quaking-induced conversion (RT-QuIC) in a relatively small proportion of sampled animals who tested positive for CWD infection by other analyses [83,84]. In addition to the detection of PrP^Sc^ in the OM, infectivity in the OM and in nasal secretions collected by nasal lavage from infected hamsters were found to be infectious by bioassay, indicating that infected animals can shed PrP^Sc^ via their NC that is potentially infectious [81]. However, these results must be interpreted with caution as the inoculations were made directly into the OBs and the retrograde transport to the OSNs in the OE may be the result of an intranerve inoculation. Additionally, it must be emphasized that the presence of PrP^Sc^ in the NC in each of the experimental studies listed above was noted only at the terminal stages of disease, and thus the period of potential shedding prior to death was relatively short.

### 4.2. Evidence from Human Prion Diseases

Several studies have identified PrP^Sc^ in the NCs of humans diagnosed with a prion disease. PrP^Sc^ was first identified in the OM collected postmortem from nine individuals who died with a diagnosis of sporadic Creutzfeldt–Jakob disease (sCJD) [85]. The presence of the prions in the OM of the NC, at the interface between the OSNs and airspace, suggested that PrP^Sc^ might be detectable in a non-invasive tissue collection procedure and perhaps provide a convenient means of antemortem diagnosis. The ability for the antemortem detection of a prion disease was subsequently demonstrated by the immunohistochemical detection of PrP^Sc^ in an olfactory biopsy collected from an individual with suspected, later confirmed, sporadic CJD [86]. The diagnostic potential for this procedure was confirmed by the application of the RT-QuIC assay on nasal brushings from individuals with both sporadic and inherited CJD [87]. RT-QuIC applied to the OM proved to be both highly specific and sensitive in the diagnosis of Creutzfeldt–Jakob disease, and the sensitivity was greater than that of the RT-QuIC analysis of cerebrospinal fluid collected from these same patients [87]. The sensitivity and specificity of RT-QuIC and PMCA in detecting PrP^Sc^ in the OM of patients with a diagnosed prion disease were confirmed in patients with fatal familial insomnia (FFI), an inherited prion disease [88]. Regarding the potential for the human NC to shed infectious prions, studies using transgenic mice have shown that OM pellets collected from sporadic CJD is infectious, but the amount of infectivity in the OM brushings was more than 10,000-fold less infectious per unit volume than that for infected brain tissue from these same patients. These findings were similar to the results of a transmission study demonstrating that PMCA-generated products from OM obtained from FFI patients inoculated into transgenic mice expressing the bank vole prion protein were infectious [89]. To date, there has been just one study of human nasal secretions (collected from the nasal vestibule), which found prion infectivity in two of 13 samples, indicating that there was lower, and in most cases undetectable, seeding activity in the nasal secretions compared to the OM nasal brushings [90]. Therefore, while the data suggest that the potential for the spread of prion diseases between humans is limited, precautions during surgical procedures involving the nasal cavity are warranted [89,90].

## 5. Summary

### 5.1. Centripetal Spread of Prions

Studies in a variety of animals have demonstrated that multiple prion strains inhaled into the NC cause disease in a relatively efficient manner. Significantly, the inhalation of infected brain homogenate into the NC has not been demonstrated to cause centripetal spread via the ON into the CNS. Instead, prions have been shown to cross the epithelial lining of the NC and enter lymphatic vessels in the lamina propria and are detected in the blood within minutes of inhalation. The specific mechanism and route of neuroinvasion following the inhalation of infectious prions has not been determined for any of the animal species examined to date. In contrast to the substantial experimental evidence for the efficient entry of prions via the NC in animals, there is no evidence that humans are infected with prions via exposure through their NC.

### 5.2. Centrifugal Spread and Shedding of Prions

Regarding the role of the NC in the centrifugal spread and shedding of prions, the evidence in experimental animals is limited and must be interpreted with caution. PrP^Sc^ was detected in the somata and dendrites of the OSNs of affected animals, and nasal secretions collected from these animals caused disease in recipient animals, but these animals had been inoculated intracerebrally or in the OBs, not by a peripheral route. In animals inoculated by a peripheral route, PrP^Sc^ was detected in OSNs but only in animals very late in the incubation period or when they demonstrated clinical signs. Thus, any shedding of prions in nasal secretions that might have taken place would only have occurred for a relatively short period of time. Centrifugally spread prions were noted in the OM of humans affected with prion disease, but the level of infectivity was very low. Therefore, while nasal brushings may be a useful diagnostic tool in humans, the evidence for the centrifugal shedding of PrP^Sc^ is limited. Taken together, the evidence indicates that the NC is a likely route of entry for prions in animals, but not humans, and that the shedding of PrP^Sc^ in nasal secretions as a significant means of transmission is not likely.

## Figures and Tables

**Table 1:** Experimental evidence for centripetal spread of prions via the nasal cavity.

Study (Year)	Species	Inoculum	Did Exposure to NC Cause Disease?	PrP^Sc^ in OE Prior to Detection in OB/Brain	Detection of PrP^Sc^ in LRS Structures
Kincaid and Bartz (2007)	Hamster	HY-bh	Yes	No	Yes
Hamir et al. (2008)	Sheep	Scrapie-bh	Yes	nd	nd
Sbriccoli (2009)	Hamster	263K-bh	Yes	No	Yes
Bessen et al. (2009)	Hamster, mice	HY or DY-bh; RML-bh	Yes; except for DY-inoculated and most LRS-deficient mice	No	Yes; except for LRS-deficient and DY-inoculated animals
Denkers et al. (2010)	Cervidized mice	CWD-bh or CWD-aerosol	Yes	No	No
Haybaeck et al. (2011)	Wild-type, transgenic, or immunodeficient mice	RML-bh or RML-aerosol	Yes	nd	Yes; except for LRS-deficient mice
Nichols et al. (2013)	Deer	CWD-bh mixed with soil	nd	nd	Yes
Denkers et al. (2013)	Deer	CWD-aerosol	Yes	nd	Yes

nd—not done; bh—brain homogenate.

**Table 2:** Evidence for centrifugal spread of PrP^Sc^ to nasal cavity.

Study (Year)	Species	Route of Exposure	Means of Detection	Location of PrP^Sc^
DeJoia et al. (2006)	Hamster	IC	IHC; IF	OM; VNE
Bessen et al. (2010)	Hamster	IC; IOB	IHC; IF; WB; RT QuIC	OM; VNE
Haley et al. (2016)	Deer	Not known	RT QuIC OM brushings	OM
Haley (2016)	Elk	Not known	RT QuIC OM brushings	OM
Zanusso et al. (2003)	Human sCJD	none	IHC; WB	OM
Tabaton et al. (2004)	Human sCJD	none	IHC; immunoblot	OM
Orru et al. (2014)	Human sCJD; inherited CJD	none	RT QuIC OM brushings	OM
Redaelli et al. (2017)	Human FFI	none	RT QuIC; PMCA	OM

IC = intracerebral; IOB = intra olfactory bulb.
